# Relationships Between Commuting and Social Capital Among Men and Women in Southern Sweden

**DOI:** 10.1177/0013916514529969

**Published:** 2015-08

**Authors:** Kristoffer Mattisson, Carita Håkansson, Kristina Jakobsson

**Affiliations:** 1 Lund University, Sweden

**Keywords:** commuting, social capital, cross-sectional design, social participation, general trust

## Abstract

The societal need for a mobile workforce increases time spent commuting and thus also the total workday. How this affects individual well-being and social life is, however, surprisingly little known. We investigated the relation between commuting time and mode, and social participation and general trust in other people as measures of social capital, using data from public health surveys conducted in 2004 and 2008 in Scania, Sweden: in all, 21,088 persons ages 18 to 65 and working at least 30 hr per week. Commuting by car was significantly associated with a higher prevalence of low social participation and low general trust compared with active commuting, and the association increased with the duration of commuting time. In contrast, public commuting was not significantly associated with decreased social capital measures except among long-duration commuters, who reported lower social participation. The overall pattern was similar for men and for women.

It has been claimed that commuting, traveling from the home to the workplace, increases material wealth (Swedish Association of Local Authorities and Regions, 2009; Swedish Government Official Report, 2007). Enlarged job regions create more opportunities for work and strengthen the economy for both individuals and society. A more flexible and accessible labor market for companies is created by making the workforce available over larger geographical areas. Individuals are given increased opportunities to find jobs and select places to live. For these reasons, there is political will in many countries to expand labor market areas, resulting in an increase in overall commuting (European Policy Brief, 2008; Swedish Association of Local Authorities and Regions, 2009). In Sweden, the average commuting distance has increased from 10 km in 1970 to 15.6 km in 2005 (The Swedish Board of Housing, Building, and Planning, 2005).

There is a concern among researchers that increased mobility in society is increasing the geographical spread of individuals’ social networks, reducing their engagement in neighborhoods and thereby threatening social welfare by lowering the sense of security and belonging on a local level (Bergström, 2010). However, there are surprisingly few data from population-based epidemiological studies exploring the individual and community effects of everyday commuting.

Social capital is a resource emerging from social relations in a society that can be used to solve problems of an individual or a collective nature (Bourdieu, 1986; Ferlander, 2007; Kawachi, Kennedy, & Glass, 1999; Putnam, 2000). “Social capital is the glue that holds societies together and without which there can be no economic growth or human well-being” (World Bank Social Capital Initiative, 1998, p. iii); it is often defined as a social network that creates benefits from the interaction between the individuals in the network (Bourdieu, 1986; Cullen & Whiteford, 2001).

According to Bourdieu (1986) and Coleman (1988), social capital exists in the structure of the social network—in contrast to economic capital, which exists in the bank, and human capital, which exists in the mind. Thus, social capital exists not in the person who possesses the resource but in that individual’s relations to other persons (Portes, 1998). Through civic participation, norms of reciprocity and trust are established, and cooperation leads to mutual benefits (Kawachi, Kennedy, Lochner, & Prothrow-Stith, 1997). Lower levels of social capital can be associated with consequences for the individual and society, such as tax evasion, low levels of political engagement, bad health, and poor educational performance (Coleman, 1988; Feng & Boyle, 2013; Putnam, 2000)

Social capital can be characterized in terms of structural and cognitive components (Cullen & Whiteford, 2001; Ferlander, 2007). The structural component consists of social networks or social participation, while the cognitive part includes norms of reciprocity and trust. The cognitive part of social capital is developed through participation in social activities and networks (Ferlander & Timms, 2001). Social networks are often considered the most important element in social capital, but without trust and norms of reciprocity the networks would collapse (Ferlander, 2007). Social capital can also be conceptualized as having both an individual and a collective level (Cullen & Whiteford, 2001; Ferlander, 2007).

According to a review article, commuting was indicated to be a stress factor interfering with living and working conditions related to both the mode of travel and to the time lost depending on the duration of the commute (Costa, Pickup, & Di Martino, 1988). Commuting may bear consequences for social capital because it prolongs the workday, and time spent commuting is time that could be spent on social participation. The mode of commuting may also affect social capital; Putnam has suggested that traveling alone by car is detrimental to the formation of social capital (Putnam, 2000). An explanation for this is that face-to-face interaction is important in the development of trust (Urry, 2002). Commuting by public transportation entails traveling with others and is therefore an arena for social interaction, which can be associated with social participation and general trust in others (Currie & Stanley, 2008). Different modes of commuting also influence the flexibility and predictability of travel between work and home (Evans, Wener, & Phillips, 2002; Lyons & Chatterjee, 2008). Disturbances in commuting can be associated with increased frustration, anxiety, and hostility, lowering social participation and general trust (Koslowsky, Kluger, & Reich, 1995).

The effects of commuting on social capital are likely to depend on the context in which the commuting takes place. However, few epidemiological studies have investigated commuting in relation to social capital, and little is known about how these associations differ across contexts as almost all previous studies have been conducted in the United Kingdom and the United States. Besser, Marcus, and Frumkin (2008) studied socially oriented trips as a measure of social capital among participants in a U.S. national health survey from 2001. A longer commuting time was significantly associated with a lack of socially oriented trips. Other studies have also found associations between longer commuting times and lower social participation (Cassidy, 1992; Flood & Barbato, 2005; Green, Hogarth, & Shackleton, 1999; Putnam, 2000). A recent U.S. study found that time spent working had no effect on political participation, whereas time spent commuting was related to a loss in political participation, especially among low-income citizens (Newman, Johnson, & Lown, 2014).

Another U.S. study conducted among automobile commuters showed that both women and men spent less time with friends as their commuting time increased (Christian, 2012). Men but not women also spent less time with family, showing that time spent commuting affects time spent on family in a gendered way. Long-distance commuting (more than 45 min) was associated with low general trust in the United States (Rahn, Yoon, Garet, Lipson, & Loflin, 2009), and in Vienna, commuting more than 30 min correlated to lower levels of social satisfaction (Delmelle, Haslauer, & Prinz, 2013).

In Sweden, most men and women ages 20 to 64 belong to the paid workforce: 88% of men and 81% of women (Statistics Sweden, 2009). Data on commuting patterns reveal important differences between men and women in commuting distance, time, and mode. In 2005, the average commuting distance was 19.1 km for men and 13.7 km for women, but the average commuting time was about 27 min for both genders, suggesting that men commute longer distances at higher speeds than women do (Sola, 2009). In 2007 in southern Sweden, commuting by car was the most common mode overall, but women were more likely than men to commute by walking, cycling, or public transportation (Indebetou & Quester, 2007). Although Swedish men and women have the same working hours on average, women in Sweden—and all over the world—perform more household work than men do (Hochschild, 1989; Sandow & Westin, 2010; Sola, 2009). Studies have shown that women who commute long distances experience more stress and time pressure than men do (Sandow, 2011). This reflects the different roles and different expectations of men and women in work life and at home that can influence the consequences of commuting in a gendered way. In a national Swedish cohort from 2000, Sandow (2013) found an association between long-distance commuting and higher separation rates. Commuting more than 45 min (one way) was associated with higher separation rate among married or registered as cohabiting couples having the same residential address. These associations differed between men and women.

The aim of the present study is to examine associations between commuting mode, commuting time and social capital, measured as social participation and generalized trust in other people, among men and women in a region where commuting is common because of a polycentric city structure. We hypothesize that more time spent commuting means less time for social participation, thereby reducing social capital, and that the mode of commuting influences social capital through both social participation and generalized trust. Moreover, we hypothesize that there may be gender differences.

## Method

### Study Area

The county of Scania in southern Sweden consists of 33 municipalities varying in size and population and covering an area of almost 11,000 km^2^; the total population is about 1.2 million and the population density is about 110 per km^2^ (Statistics Sweden, 2011). The largest city is Malmö, which had 258,000 inhabitants in 2005 (Statistics Sweden, 2011). Most parts of Scania are experiencing an increase in population, especially in the southwest around Malmö and Lund (Scania Regional Council, 2009). Job opportunities are also concentrated in the southwest, but even so, Scania is considered polycentric, having important emerging centers in Helsingborg and Kristianstad, and political will exists to support and develop this societal structure (Scania Regional Council, 2011).

During the last decade, there has been a large increase in commuting, partly explained by the building of the Öresund Bridge to Denmark. The number of jobs that can be reached in 30 min is much higher when traveling by car from most places than it is when traveling by public transportation (Scania Regional Council, 2009).

### Survey Participants

This cross-sectional study is based on a retrospective data set from two population-based public health questionnaires from 2004 and 2008 (Rosvall, Grahn, Modén, & Merlo, 2009; Rosvall, Khan, Nilsson, & Ostergren, 2005), from which the study population has been drawn. From each municipality, 200 men and 200 women aged 18 to 80 years were randomly selected (considered to be one sampling unit). The larger cities were, however, subdivided into multiple sampling units. The overall response rate was 59% in 2004 and 54.1% in 2008. Overlap among participants in the two different survey years is negligible. For the present study, respondents who had answered questions about commuting, who were working more than 30 hr per week, and who were less than 65 years old were selected to form the study population (*n* = 21,088; 12,184 in 2004 and 8,904 in 2008; Table 1). The questionnaires consisted of more than 100 questions intended to increase knowledge about public health in Scania. The content of the questionnaires administered in 2004 and in 2008 was very similar, and the questions included in this study were identical. Health-related outcomes in relation to commuting have previously been reported (Hansson, Mattisson, Björk, Östergren, & Jakobsson, 2011).

**Table 1.** Characteristics of Men and Women Study Participants by Commuting Mode and Time.

	Car (min)			Public (min)			Active (min)	Total
Characteristic	<30	30-60	>60	<30	30-60	>60	<30	
*Men (N)*	*5,755*	*1,438*	*339*	*487*	*503*	*291*	*1,827*	*10,640*
Low social participation (%)	34	32	38	25	21	26	30	32
Low general trust (%)	35	32	38	31	27	31	32	33
Age (median years)	45	47	48	40	42	41	45	45
University education (%)	32	37	41	54	63	65	43	38
Manual workers (%)	41	35	34	30	22	20	38	38
Born abroad (%)	6	3	3	9	6	7	7	6
Urban residents (%)	21	11	16	38	34	36	42	25
Cohabiting with partner (%)	79	84	85	75	79	77	74	79
Living with children under 13 years (%)	34	31	32	34	36	31	27	32
Job strain (%)	20	18	19	20	17	22	18	19
Income (median number of price base amounts)	7.4	7.8	8.6	7.0	7.6	7.5	6.9	7.4
Ever having problems paying bills (%)	21	19	24	24	24	25	19	21
History of unemployment (%)	8	9	12	13	13	16	10	10
Often working overtime (%)	29	29	38	24	21	30	27	28
*Women (N)*	*5,000*	*842*	*110*	*845*	*809*	*293*	*2,549*	*10,448*
Low social participation (%)	29	27	19	27	25	27	25	27
Low general trust (%)	36	32	34	38	32	33	33	35
Age (median years)	45	44	44	44	45	42	47	45
University education (%)	44	54	63	44	56	62	50	48
Manual workers (%)	31	19	12	34	21	20	34	30
Born abroad (%)	5	4	5	10	7	9	6	6
Urban residents (%)	18	14	13	36	33	39	45	27
Cohabiting with partner (%)	81	82	72	70	76	73	71	77
Living with children under 13 years (%)	35	35	25	27	25	18	22	30
Job strain (%)	24	23	22	27	28	27	25	25
Income (median number of price base amounts)	5.7	6.1	7.3	5.5	5.8	5.5	5.6	5.7
Ever having problems paying bills (%)	23	23	20	27	23	26	21	23
History of unemployment (%)	9	11	15	12	14	21	8	10
Often working overtime (%)	22	26	36	17	21	25	22	22

*Source*. Public Health Surveys 2004 and 2008, and authors’ calculations. Active <30 min is the reference group in the Poisson regression.

### Exposure Variables

Information on commuting time was obtained through the question “How much time does it take to get to work (single journey)?” The six response alternatives were *less than 15 min, 15 to 30 min, 30 to 60 min, 1 to 1.5 hr, 1.5 to 2 hr*, and *more than 2 hr*. Responses were recoded into three categories: (a) less than 30 min, (b) 30 to 60 min, and (c) more than 1 hr.

Commuting mode was coded from responses to the question “How do you usually get to work?” The response alternatives to this question were traveling by *walking, biking, car, bus, train*, and *other*. Multiple alternatives could be chosen. The responses were recoded into three categories: (a) active, (b) car, and (c) public transportation. *Active* commuting included only walking or biking responses. *Car* included everyone who responded using a car only or a car combined with walking or biking. *Public* included all those who reported traveling by bus or train, regardless of other modes reported. The alternative *other* was not included in the classification. This classification was made to group respondents based on the flexibility of the modes, active being the most flexible and public the least flexible in terms of schedules and routes.

The reclassified time and mode variables were combined into a commuting exposure variable with seven categories (one for active, three for car commuting, and three for public transportation; Hansson et al., 2011). The reference category was active commuters and included those who reported commuting times under 30 min by walking or cycling. Respondents who commuted more than 30 min by walking or biking were excluded because there were very few of them.

### Outcome Variables

Social participation represents the structural part of social capital. Social participation was measured with a multiple-choice question asking whether, during the last 12 months, respondents had participated in a range of listed activities (Table 2); the question measures how a person takes part in formal and informal groups in society. The variable was dichotomized for *high* (more than three reported activities) and *low social participation* (three activities or fewer; Lindstrom, Merlo, & Ostergren, 2002). This question has been used as a measure for social participation in Sweden since the 1960s and has been included in all public health surveys conducted in Scania (The National Central Bureau of Statistics, 1980). The measure has been validated in earlier studies (Hanson, Ostergren, Elmstahl, Isacsson, & Ranstam, 1997).

**Table 2.** Social Capital Variables (Variable and Question).

Social participation	
Question: Have you during the last 12 months:	Recoding
Participated in a study circle/course at your workplace?	Low social participation = any three or fewer activities
Participated in a study circle/course in your spare time?	
Participated in a union meeting?	High social participation = any four activities or more
Participated in a meeting of another association?	
Been to the theater/cinema?	(The alternative “None of the above” was not included in the recoding)
Been to an art exhibition?	
Participated in a religious meeting?	
Been to a sporting event?	
Sent a letter to a newspaper?	
Participated in a demonstration?	
Visited a public place—for example, a night club or a dance club or similar place?	
Participated in a larger family gathering?	
Been to a private party?	
(None of the above)	
General trust	
Question: You can trust most people.	Recoding
Do not agree at all	Low general trust = Do not agree at all or Do not agree
Do not agree	
Agree	High general trust = Agree or agree completely
Agree completely	

*Source*. Public Health Surveys 2004 and 2008, authors’ recoding.

Generalized trust of other people was used to describe the cognitive part of social capital. It was measured with the question, “Can you trust most people?” (Table 2). This variable was also dichotomized. The responses *agree* and *agree completely* were coded as high general trust. *Don’t agree* and *don’t agree at all* were coded as low general trust. Social capital measured as social participation and general trust in other people has been used in several other studies (Giordano, Bjork, & Lindstrom, 2012).

### Covariates

Covariates were selected based on theoretical relationships to both the outcome variables and the exposure variable, and were added to the crude model in two steps. The first step was a partially adjusted model including primary covariates that describe fundamental demographic and socioeconomic characteristics, such as age, country of origin, occupational status, and educational status. These covariates are stable and to a large extent are set before the person starts to commute, and we presume that they precede the commuting pattern—that is, they exhibit a unidirectional association. Participants were grouped into four categories based on age (18-34, 35-44, 45-54, and 55-64) and three categories based on education (9 years or less, 10-12 years, and 13 years or more). The country of origin variable grouped those born in Sweden, the Nordic countries, North America, and Australia together, while all others were grouped into a second category. Occupational class was represented by six groups using the Swedish socioeconomic classification (unskilled manual laborers, skilled manual laborers, farmers and entrepreneurs, low-level non-manual workers, medium-level non-manual workers, and high-level non-manual workers; Statistics Sweden, 2013).

In the second step the model was fully adjusted, including covariates describing current life and work situation. For these variables, the associations with commuting pattern may be bidirectional, in contrast to the covariates in the first step. These covariates included living in an urban area, job strain, family situation, income, history of unemployment, having problems paying bills, and working overtime. People living in the four largest cities in Scania (Malmö, Lund, Helsingborg, and Kristianstad) were classified as living in urban areas. Those classified as having both high job demands and low job control, based on a Swedish translation of the job content questionnaire (Karasek et al., 1998), were considered to experience job strain. Family situation was assessed based on questions about whether the person was living with children in the age ranges 0-6, 7-12, 13-17, and 18 or above and sharing a household with parent–sibling, husband–wife, cohabiting partner, or other adults. Each category was treated as a separate dichotomous variable.

### Statistical Analysis

Poisson regression was used to analyze associations between social capital and commuting owing to a high prevalence of the two outcome variables. The use of Poisson regression instead of logistic regression avoids overestimation of the prevalence ratios (Barros & Hirakata, 2003). Estimates of prevalence ratios were obtained from generalized linear models using the Poisson distribution with the natural logarithm as link function and using robust variance estimator. This is a corrected model-based estimator that provides a consistent estimate of the covariance, making the statistical inference more robust to incorrect specification of the variance (e.g., if the variance exceeds the mean) and link function. Prevalence ratios and 95% confidence intervals were calculated for the crude, partially adjusted, and fully adjusted models for men and women separately and for the total sample.

The crude model included the exposure variable (commuting mode and time) and no covariates. The partially adjusted model included the primary covariates described above. In the fully adjusted model, all covariates from the partially adjusted model were included, along with the variables describing life situation and work.

Every single covariate was also tested one by one. Almost all of the covariates were statistically significant for both men and women. Alternative models including only statistically significant covariates were also explored, but the results were not altered. We also checked for calender year effects, but this did not alter the results.

All analyses were performed in the total sample and also stratified by gender. Differences between men and women were evaluated by including a multiplicative interaction variable between gender and commuting.

## Results

Descriptive data are displayed in Table 1. The most common daily commute for both men and women was by car less than 30 min. The second most common commute was by active transport, and the least common was public transport. More women than men commuted actively or by public transport less than 60 min. More men than women commuted by car. Women and men living in urban areas, however, were both more likely to commute by public transportation.

The covariate pattern differed between men and women. Overall, women were better educated and experienced higher job strain than men, while men worked more in manual jobs and had higher incomes than women. Women with longer commuting times were well educated and had higher incomes except for public commuters, for whom income was about the same for men and women regardless of commuting time.

Low social participation was reported for 27% of women and 32% of men. Low general trust was reported by 35% of women and 33% of men. Younger persons had higher social participation and lower general trust than older participants did. People with higher levels of education and in non-manual work had higher levels of social participation and general trust.

Women commuting by car more than 60 min had the highest social participation (19% low social participation), and men commuting by car more than 60 min had the lowest social participation (38% low social participation). According to the Poisson regression model, all car commuters reported a lower level of social participation than active commuters did (Table 3). The lowest social participation was seen among car commuters commuting more than 60 min; a similar finding was observed for low general trust (Table 4). Public commuters traveling more than 60 min had a higher estimate of low social participation than active commuters did, but differences were not statistically significant. No difference in the prevalence of general trust in other people was found between public commuters and active commuters. Overall, the prevalence ratios from the regression models were relatively stable for the crude, partially, and fully adjusted models.

**Table 3.** Prevalence Ratios for Low Social Participation for Men and Women From Poisson Regression With Commuting Mode and Time.

	Total		Women		Men	
Regression model	*N*	Prevalence ratio [95% CI]	*N*	Prevalence ratio [95% CI]	*N*	Prevalence ratio [95% CI]
Crude						
Active (<30 min)	4,361	Reference	2,543	Reference	1,818	Reference
Car (<30 min)	10,699	**1.14 [1.08, 1.21]**	4,972	**1.13 [1.04, 1.22]**	5,727	**1.12 [1.03, 1.21]**
Car (30**-**60 min)	2,267	**1.09 [1.01, 1.19]**	838	1.06 [0.93, 1.20]	1,429	1.05 [0.95, 1.17]
Car (>60 min)	444	**1.23 [1.07, 1.42]**	108	0.77 [0.52, 1.14]	336	**1.26 [1.09, 1.48]**
Public (<30 min)	1,329	0.97 [0.87, 1.07]	843	1.07 [0.94, 1.22]	486	**0.84 [0.71, 0.99]**
Public (30-60 min)	1,308	**0.86 [0.77, 0.96]**	807	0.99 [0.87, 1.14]	501	**0.69 [0.58, 0.83]**
Public (>60 min)	581	0.98 [0.85, 1.13]	290	1.07 [0.88, 1.32]	291	0.87 [0.71, 1.07]
Partially adjusted						
Active (<30 min)	4,348	Reference	2,539	Reference	1,809	Reference
Car (<30 min)	10,678	**1.10 [1.04, 1.16]**	4,967	**1.13 [1.04, 1.22]**	5,711	**1.08 [1.00, 1.16]**
Car (30**-**60 min)	2,254	**1.14 [1.05, 1.23]**	833	**1.23 [1.08, 1.39]**	1,421	1.08 [0.97, 1.19]
Car (>60 min)	435	**1.25 [1.10, 1.44]**	106	1.02 [0.69, 1.50]	329	**1.26 [1.09, 1.46]**
Public (<30 min)	1,321	1.02 [0.93, 1.13]	842	1.03 [0.91, 1.17]	479	0.99 [0.85, 1.17]
Public (30**-**60 min)	1,302	1.05 [0.95, 1.17]	806	1.13 [0.99, 1.29]	496	0.93 [0.78, 1.11]
Public (>60 min)	570	**1.25 [1.09, 1.43]**	286	**1.26 [1.04, 1.52]**	284	1.20 [0.99, 1.46]
Fully adjusted						
Active (<30 min)	4,130	Reference	2,415	Reference	1,715	Reference
Car (<30 min)	10,228	**1.09 [1.03, 1.15]**	4,710	**1.10 [1.01, 1.19]**	5,518	**1.08 [1.00, 1.16]**
Car (30-60 min)	2,149	**1.12 [1.03, 1.21]**	792	**1.17 [1.03, 1.34]**	1,357	1.07 [0.96, 1.18]
Car (>60 min)	385	**1.28 [1.11, 1.48]**	93	0.97 [0.63, 1.50]	292	**1.31 [1.12, 1.53]**
Public (<30 min)	1,245	0.99 [0.90, 1.09]	793	1.00 [0.88, 1.13]	452	0.96 [0.82, 1.13]
Public (30**-**60 min)	1,214	1.00 [0.90, 1.12]	747	1.07 [0.94, 1.23]	467	0.87 [0.73, 1.05]
Public (>60 min)	504	1.15 [1.00, 1.34]	253	1.21 [0.99, 1.47]	251	1.08 [0.87, 1.35]

*Note*. This multivariate analysis has controlled for covariates in two steps. The partially adjusted model controlled for age, place of birth, occupational class, and educational level. The fully adjusted model also controlled for job strain, unemployment during the last 3 years, overtime work, income, financial stress, residential location, and family situation. CI = confidence interval. Bold text marks significance at the 5% level.

**Table 4.** Prevalence Ratios for Low General Trust for Men and Women From Poisson Regression With Commuting Mode and Time.

	Total		Women		Men	
Regression model	*N*	Prevalence ratio [95% CI]	*N*	Prevalence ratio [95% CI]	*N*	Prevalence ratio [95% CI]
Crude						
Active (<30 min)	4,337	Reference	2,529	Reference	1,808	Reference
Car (<30 min)	10,664	**1.08 [1.03, 1.14]**	4,955	**1.09 [1.02, 1.17]**	5,709	**1.09 [1.01, 1.17]**
Car (30**-**60 min)	2,262	0.98 [0.91, 1.05]	833	0.97 [0.86, 1.08]	1,429	1.00 [0.90, 1.10]
Car (>60 min)	440	1.12 [0.98, 1.27]	107	1.01 [0.77, 1.32]	333	**1.18 [1.01, 1.37]**
Public (<30 min)	1,322	1.07 [0.99, 1.17]	840	**1.13 [1.02, 1.25]**	482	0.96 [0.83, 1.12]
Public (30-60 min)	1,300	0.91 [0.83, 1.00]	801	0.96 [0.85, 1.07]	499	**0.84 [0.71, 0.98]**
Public (>60 min)	579	0.96 [0.85, 1.09]	289	0.97 [0.82, 1.16]	290	0.96 [0.80, 1.16]
Partially adjusted						
Active (<30 min)	4,324	Reference	2,525	Reference	1,799	Reference
Car (<30 min)	10,642	**1.06 [1.01, 1.11]**	4,95	**1.07 [1.01, 1.15]**	5,692	1.04 [0.97, 1.12]
Car (30**-**60 min)	2,250	1.04 [0.97, 1.12]	828	1.04 [0.93, 1.16]	1,422	1.04 [0.94, 1.15]
Car (>60 min)	431	**1.23 [1.08, 1.41]**	105	1.20 [0.92, 1.56]	326	**1.23 [1.06, 1.44]**
Public (<30 min)	1,314	1.05 [0.97, 1.14]	839	1.06 [0.96, 1.17]	475	1.03 [0.89, 1.19]
Public (30**-**60 min)	1,294	0.99 [0.90, 1.08]	800	0.99 [0.89, 1.11]	494	0.97 [0.83, 1.14]
Public (>60 min)	568	1.09 [0.96, 1.23]	285	1.03 [0.87, 1.22]	283	1.15 [0.96, 1.39]
Fully adjusted						
Active (<30 min)	4,110	Reference	2,404	Reference	1,706	Reference
Car (<30 min)	10,198	**1.09 [1.04, 1.15]**	4,696	**1.10 [1.03, 1.18]**	5,502	**1.09 [1.01, 1.18]**
Car (30**-**60 min)	2,147	**1.08 [1.00, 1.17]**	788	1.06 [0.95, 1.19]	1,359	1.10 [0.99, 1.22]
Car (>60 min)	382	**1.31 [1.14, 1.50]**	92	1.29 [0.98, 1.70]	290	**1.31 [1.12, 1.54]**
Public (<30 min)	1,238	1.03 [0.95, 1.12]	790	1.04 [0.94, 1.15]	448	1.01 [0.87, 1.18]
Public (30**-**60 min)	1,207	0.97 [0.88, 1.06]	742	0.95 [0.85, 1.07]	465	0.97 [0.83, 1.14]
Public (>60 min)	501	1.02 [0.89, 1.16]	251	0.96 [0.80, 1.15]	250	1.08 [0.89, 1.32]

*Note*. This multivariate analysis has controlled for covariates in two steps. The partially adjusted model controlled for age, place of birth, occupational class, and educational level. The fully adjusted model also controlled for job strain, unemployment during the last 3 years, overtime work, income, financial stress, residential location, and family situation. CI = confidence interval. Bold text marks significance at the 5% level.

Separate models for men and women are also presented in Tables 3 and 4. Overall, the patterns were rather similar, and a multiplicative interaction variable for gender and commuting was not statistically significant for low social participation (*p* = .41) or for low general trust (*p* = .98). However, it should be noted that female car commuters traveling more than 60 min had no increased prevalence of low participation, in contrast to their male counterparts. The opposite finding was observed among female public commuters traveling more than 60 min, who had a higher prevalence of low participation than their male counterparts did.

## Discussion

Commuting by car, compared with active commuting, was associated with low social participation and low general trust. Prevalence rates increased with the duration of the car commute. In contrast, commuting by public transportation was not associated with low social participation or low general trust, except among long-duration public commuters, who reported lower social participation. Men and women exhibited markedly different commuting patterns, but the differences between men and women with respect to associations between social capital and commuting were, overall, not statistically significant.

One strength of this study is the large number of participants sampled from the general population, coupled with the comprehensive information about possible covariates. This makes it possible to control for a large number of covariates, thus increasing the generalizability of the results. Even so, it is important to consider that commuting is context-dependent and results should therefore only be generalized with caution. The exposure experienced during the commute is complex, as both a loss of time that commuters are free to spend on other activities and psychological and physiological reactions to the commute occur. The analysis of time and mode can to a certain extent elucidate these aspects, but a drawback is the lack of information on the subjects’ perceptions of their commutes.

The reason for each commuter choosing the mode of commuting is complex and includes both environmental or structural factors, such as physical neighborhood and labor market structure, in combination with each individual’s situation in terms of gender, income, household composition, and personality (Schwanen & Mokhtarian, 2005). In our study, access to a wealth of covariate information allowed adjustment on fundamental demographic and socioeconomic factors, as well as additional, more specific factors related to work, home, and family conditions, but the cross-sectional design inevitably excludes explorations of causality. Moreover, we had no information on personality traits and no contextual information except a classification of the home location as either urban or rural.

Looking at both the structural part and the cognitive part of social capital is a new perspective in the present study; previous studies on commuting and social capital have not used this perspective to our knowledge. The question used as a measure of social participation has long been considered relevant in the Swedish context (Lindstrom, Moghaddassi, & Merlo, 2003). Clearly, changes in social participation have occurred since the items were developed, and new activities have emerged, such as the use of social media and online social networks as means to maintaining individual social capital (Johnston, Tanner, Lalla, & Kawalski, 2013), and physical exercise as a form of social activity. However, as the surveys were performed in 2004 and 2008—that is, before the use of social media increased so markedly—we believe that the lack of a list of “updated” social activities for construction of social participation is a minor drawback.

Putnam (2000) has suggested that time lost to travel has negative consequences for social capital formation. This appears in our study as the increasing prevalence of low social participation with the increasing duration of car commuting and is also indicated among public commuters with the longest commutes. Likewise, lower levels of satisfaction with social contacts were reported among commuters (more than 30 min) in Vienna (Delmelle et al., 2013), as well as lower prevalence of political participation in lower-income long-duration commuters in the United States (Newman et al., 2014). This has also been reported in earlier studies conducted in the United States. Commuters with longer travel times or distances spent less time socializing and therefore experienced disruptions in their social lives (Cassidy, 1992). A national study investigated the number of socially oriented trips a person takes as a measure of social capital and used commuting time as the exposure (Besser et al., 2008). The study found that every 10-min increase in commuting time was associated with a 4.1% increase in the odds of making no socially oriented trips. Another study showed that long-duration commutes (over 45 min) were associated with low general trust (Rahn et al., 2009).

The lower social participation and general trust among car commuters compared with active and public commuters independent of commuting time (except among women commuting long durations) in the present study accord with Putnam’s suggestions that car dependency decreases social capital (Putnam, 2000). Similarly, it has been observed that political participation was less prevalent in U.S. communities attesting a high prevalence of single-car commuters and higher in communities with walking, biking, and public transit (Hopkins & Williamson, 2014).

If the commute is experienced as disturbing, the person’s mood can be affected negatively in the form of tension, irritability, or fatigue (Kluger, 1998; Novaco, Stokols, & Milanesi, 1990). Increased stress levels might reduce one’s will to interact socially and thereby affect social participation and general trust. A tentative explanation for the higher prevalence of low social participation observed among car commuters in our study is that car users consider their journeys more stressful than active and public commuters consider theirs. This was seen in a study conducted among university employees in the United Kingdom, where car commuters experienced more stress than public and active commuters did (Gatersleben & Uzzell, 2007). In another study, single drivers experience more of this negative stress than people commuting by car with others (Schaeffer, Street, Singer, & Baum, 1988). Yet another study showed that car commuters experienced more negative effects on mood than train commuters did (Wener & Evans, 2011). However, in a previous study in our study population, car commuters did not experience higher risks of everyday stress than public transportation commuters did (Hansson et al., 2011). Thus, differences in travel-related stress levels are a less likely explanation for the different patterns between car and public transportation commuting and social participation and general trust observed in our study. Public transportation, by definition, means traveling with others, and social interaction during the commute is thereby facilitated (Currie & Stanley, 2008). Social interaction that occurs while commuting by public transportation is not likely to involve close relationships but, rather, more superficial interaction with people outside the individual’s common social circle (Currie & Stanley, 2008). This creates the opportunity to form so-called bridging social capital. It is also possible that some people with an inclination to lower levels of social participation choose to commute by car.

There were marked differences between commuting patterns for men and women also with respect to covariates. The associations between commuting patterns and social capital were generally not statistically different between men and women, when tested with a multiplicative interaction term. Still, some observations that warrant further consideration were seen. A clearly divergent group—albeit small—comprised female long-distance car commuters (more than 60 min) who demonstrated no increased prevalence of low social participation. A high percentage of this group was university educated and exhibited a low level of manual work, and few of whom were living with a partner or children below age 13. Presumably this group is career-oriented women with few family obligations—all in contrast to the men commuting more than 60 min. Furthermore, the difference between female and male long-duration (more than 60 min) public commuters should be noted: Women but not men exhibited a low prevalence of social participation.

The characterization of the commute in this study was limited to time and mode. In further research, commuting in relation to social capital should be studied longitudinally and in more detail, taking into account the flexibility and uncertainty in commuting, commuters’ attitudes toward the commute, and their reason for having to commute. It would be especially interesting to examine the link between the stress experienced during the commute and social capital; we believe this to be an important factor leading to lower levels of social capital and to greater loss of time that could be spent on social participation. Thus, both a quantity-focused “resources” model, in which commuting entails less free time, as well as a “commuter’s strain” model, which emphasizes time spent in transit as a psychological or physiological burden over and above the workday, should be explored.

This study has focused on social capital measured on an individual level, but social capital can also be explained and measured on its societal level. Active and public transport can enhance the livability of a city and create an environment that is better suited to social activities (Vuchic, 1999). If certain types of commuters tend to live together, this could affect individual social participation; it could also thereby influence the social capital from a societal perspective. When planning public transportation, it is important to not exclude certain groups, such as people with low socioeconomic status or people with disabilities (Currie & Stanley, 2008). Poor planning can lead to social isolation for these groups.

The main finding in this study from Scania, where 70% of men and 57% of women commute by car, is that car commuting was associated with lower levels of social participation and general trust. Social capital, like economic and cultural capital, can be seen as a resource for society. It would therefore be beneficial in urban planning to explore the balance of positive and negative effects on an individual and on a societal level when increasing the geographical sizes of labor markets and thus also increasing the duration of commutes for the population. Finding this balance would help both individuals and society to create a more sustainable existence in which work–life conflicts can be minimized.
